# BASE Jumping in the Lauterbrunnen Valley: A Retrospective Cohort Study from 2007 to 2016

**DOI:** 10.3390/ijerph20043214

**Published:** 2023-02-12

**Authors:** Monika Brodmann Maeder, Simon Andenmatten, Jasmin Sumiko Lienert, Thomas Von Wyl, Aristomenis K. Exadaktylos

**Affiliations:** 1 Department of Emergency Medicine, Bern University Hospital, Bern University, 3010 Bern, Switzerland; 2 Institute of Mountain Emergency Medicine, EURAC Research, 39100 Bolzano, Italy; 3 Hôpital Cantonal de Fribourg, 1708 Fribourg, Switzerland; 4 Department of Anesthesia and Critical Care, Hospital Interlaken, 3800 Interlaken, Switzerland

**Keywords:** BASE Jumping, extreme sports, trauma, evacuation, emergency medicine, NACA, ISS

## Abstract

Background: BASE jumping, and especially BASE jumping with the help of wingsuits, is considered one of the most dangerous airborne sports. The valley of Lauterbrunnen in Switzerland has become infamous for the large number of BASE jumps and the high rate of accidents and fatalities. The aim of this study was to evaluate the morbidity and mortality of BASE jumping, to determine the severity of injuries and injury patterns of BASE jumping accidents and to compare preclinical assessment with clinical diagnoses to detect under- or overtriage. Methods: This retrospective, descriptive cohort study covers a period of 10 years (2007–2016). The evaluation covered all BASE jumping incidents in the valley of Lauterbrunnen that required either a helicopter mission by the local HEMS (Helicopter Emergency Medical Service) company of Lauterbrunnen, Air Glaciers, or medical care in the regional hospital, the level I trauma centre or the medical practice of the local general practitioner. Besides demographic data, experience in BASE jumping and skydiving as well as BASE jumping technique(s) and details about the rescue missions were collected. The medical data focused on the severity of injuries, as expressed by the National Advisory Committee of Aeronautics (NACA) score in the prehospital assessment as well as the Abbreviated Injury Scale (AIS) and Injury Severity Score (ISS) retrieved from the clinical records in the hospital or medical practice setting. Results: The patients were predominantly young, experienced male BASE jumpers. Morbidity (injury risk) ranged from 0.05% to 0.2%, and fatality risk from 0.02% to 0.08%. Undertriage was low, with only two cases. Overtriage was significant, with 73.2% of all NACA 4–6 cases not qualifying for major trauma. Conclusions: BASE jumping remains a high-risk sport and is associated with significant rates of injuries and fatalities. Comparison with previous studies indicated that the injury rate may have decreased, but the fatality rate had not. In this known BASE jumping environment, prehospital assessment appears to be good, as we found a low undertriage rate. The high overtriage rate might be an expression of physicians’ awareness of high-velocity trauma mechanisms and possible deceleration injuries.

## 1. Introduction

Jumping from buildings, antennas, spans, or the earth with a parachute (BASE jumping) is an increasingly popular high-risk sport. BASE jumping has become very popular since the first enthusiasts started in the late 1970s. In 2016, 2000 BASE jumpers were registered worldwide on a private website [1].

BASE jumping, and especially BASE jumping with the help of wingsuits, is considered one of the most dangerous airborne sports [2]. Wingsuits are special jumpsuits with “wings” expanding between the extremities and inflatable pressurised cells. Another type of suit used are tracksuits, which also inflate, but maintain the separation of arms and legs. These suits allow BASE jumpers to ‘fly’ at a lower descent rate with a longer time in free fall and higher speed. BASE jumpers in general ‘fly’ with high velocity. It is mainly the wingsuit that enables them to fly at a speed of more than 250 km/h. When a human body hits obstacles at this speed, survival is improbable [3,4]. Other trauma mechanisms are wall impacts during the start, hard landings or landing in trees [4]. Injury severity is more likely to be underestimated than overestimated in BASE jumping injuries, given the challenges related to the diagnosis of deceleration injuries [5,6]. This fact can result in undertriage, i.e., inaccurate triage of patients not transported to a trauma centre, even though they required a high level of care [7]. Publications on BASE jumping tend to concentrate on mortality, have low case numbers, or rely on data retrieved from BASE jumpers themselves [4,8,9]. A publication from the Kjerag massif in Norway estimated a risk of 0.4% for any injury and 0.04% for fatalities in BASE jumping [2]. In another study, the same authors estimated that the risk for an injury in BASE jumping was 5–8 times higher than in skydiving [10].

The valley of Lauterbrunnen is a famous site for BASE jumping and attracts athletes from all over the world. This is not only because the valley has a very attractive landscape but also because, unlike in many other places, BASE jumping has never been banned. According to unofficial information, the valley of Lauterbrunnen registered around 5000 jumps per year at the beginning of BASE jumping in the early 2000s and about 20,000 BASE jumps per year in 2016 [11]. In the BASE Fatality List [12], Switzerland has the highest number of fatalities worldwide, with 100 of 419 fatalities since 1981; 62 of these 100 fatalities are related to the use of wingsuits. Most of these accidents happened in the valley of Lauterbrunnen, which has become infamous for the large number of BASE jumps and the high rate of accidents and fatalities.

Most of the evacuations for uninjured and injured BASE jumpers take place in remote areas, where helicopter missions are often necessary. The local HEMS company, Air Glaciers, performs rescue missions almost exclusively for BASE jumpers in the valley of Lauterbrunnen; these rescue missions are often technically challenging. For several years, the chief helicopter emergency physician, who had his private practice in Lauterbrunnen, treated patients with minor injuries in his private practice. Almost all other accidents triggered a rescue mission by helicopter and referral to either the regional hospital in Interlaken or the level I trauma centre in Berne. The hospital of Interlaken, a secondary-care hospital located less than 20 km from Lauterbrunnen, can provide care for patients with mild to moderate injuries. All severe trauma cases are referred to the level I trauma centre in the University Hospital in Berne, a tertiary-care hospital. By retrieving information from the helicopter rescue company, together with the list of all known incidents related to BASE jumping held by the local general practitioner and the two hospitals, we were able to identify most evacuations of uninjured BASE jumpers and referrals of injured patients to the general practitioner and the two hospitals.

The aim of the present retrospective cohort study was to evaluate morbidity and mortality of BASE jumping in the valley of Lauterbrunnen, and to determine the severity of injuries and injury patterns of BASE jumping accidents with regard to the mechanism of injury. Moreover, we aimed to compare preclinical assessment with clinical diagnoses in order to find under- or overtriage. The Bern Ethical Committee (Kantonale Ethikkommission Bern) approved the study and waived informed consent, stating that “The research project is a reuse of biological material and health-related personal data in the absence of consent.” (KEK Nr. 2018-01195)

## 2. Materials and Methods

This retrospective, descriptive cohort study covers a period of 10 years (2007–2016). We evaluated all BASE jumping incidents in the valley of Lauterbrunnen that provoked a helicopter mission by Air Glaciers, the local HEMS company, or medical care in the medical practice of the local general practitioner.

We cross-referenced a database of admissions for BASE jumping incidents in the two referring hospitals with an unofficial register of all known BASE jumping incidents maintained by Dr Bruno Durrer, general practitioner in Lauterbrunnen and until 2016 chief emergency physician at Air Glaciers in Lauterbrunnen, and with the BASE fatality list, an up-to-date list of all known casualties in BASE jumping worldwide, as available online [12].

The following data were recorded: demographic data (age at the time of the accident, sex, nationality), experience in BASE jumping and skydiving, expressed as number of flights before the accident, BASE jumping technique(s) and details about the rescue mission. Medical data focused on the severity of injuries, as expressed by the National Advisory Committee of Aeronautics (NACA) score in the prehospital assessment as well as the Abbreviated Injury Scale (AIS) and Injury Severity Score (ISS) retrieved from the clinical records in the hospital or medical practice setting.

The NACA score is a scoring system widely used to assess the severity of medical emergencies in the preclinical setting [13,14,15] on a scale of 0 to 7, the minimum being 0 (unharmed) and the maximum 7 (deceased). An NACA score of >3 describes a potentially life-threatening health issue [13,16]. The ISS is an established medical score to assess trauma severity [17]. It is calculated from the AIS, an anatomy-based coding system created by the Association for the Advancement of Automotive Medicine to classify and describe the severity of injuries [18]. ISS > 15 is generally considered a major trauma, even though this definition has been questioned lately [19,20]. In this study, we used the 2005 Update 2008 version of the AIS [18].

**Figure 1:**
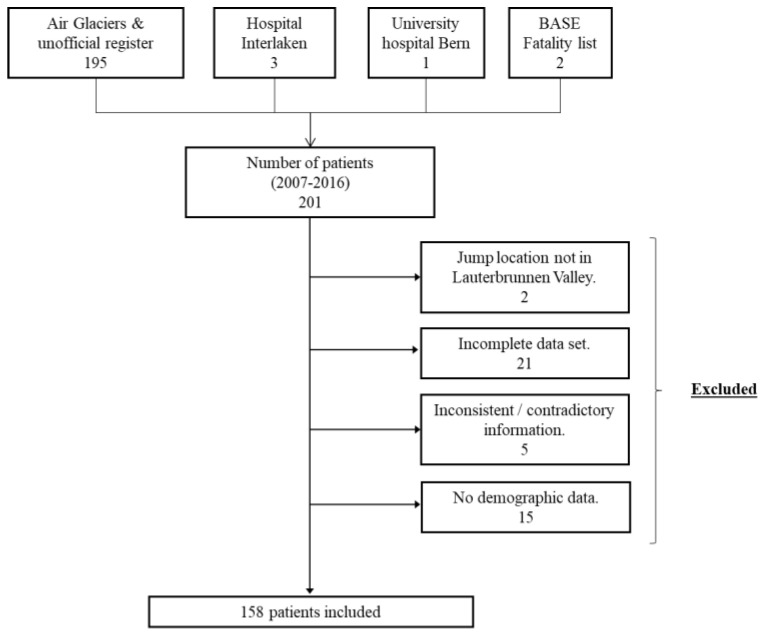
Study design.

Exclusion criteria were jump location other than in the valley of Lauterbrunnen, very incomplete data set (no or incomplete medical report, no NACA score, missing demographic data), and inconsistent or contradictory information. This resulted in the inclusion of 158 patients (Figure 1).

We calculated the risk of BASE jumping expressed as the number of injuries (NACA 1–6) per number of BASE jumps in the valley of Lauterbrunnen and the number of fatalities (NACA 7 or deceased in the hospital) per number of jumps. Although a yearly landing card system is in use in the municipal area of Lauterbrunnen, the number of jumps realised did not have to be disclosed at that time. Therefore, no accurate information of jump numbers could be obtained from the Swiss Base Association (SBA). The presidents of the SBA estimate the number of jumps to have been around 5000 in 2006 and around 20,000 in 2016 [11]. 2006 marks the beginning of BASE jumping in Lauterbrunnen, and the rising numbers are due to the increasing popularity of the sport. Morbidity and mortality were therefore expressed as ranges for injuries and fatalities between 5000 and 20,000 jumps/year.

**Figure 2:**
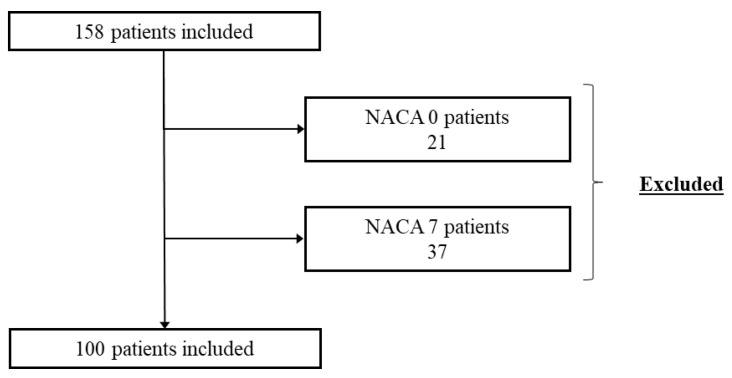
Overview of NACA scores.

As a secondary outcome, we calculated the mean ISS for each category of NACA score, excluding NACA 0 (uninjured, evacuation only) and NACA 7 (no forensic data available to calculate ISS) (Figure 2).

Overtriage was expressed as the proportion of patients without severe injuries (ISS < 16) but with NACA 4–6; undertriage was expressed as the number of patients with ISS > 15 but with NACA 1–3. Additionally, we assessed reasons for interhospital transfers from the local hospital to the level I trauma centre. All methods were performed in accordance with the relevant guidelines and regulations.

## 3. Results

Our study included 158 patients from 2007 to 2016 with a mean age of 34.3 (range 19–57), most jumpers being males (147, 93%). A wide distribution of nationalities was found, with six countries counting for 62% (n = 98) of all BASE incidents (USA 31; France 18; Germany 14; Australia 13; Switzerland 12; and the United Kingdom 10).

In our data, mostly wingsuits (44.9%, 71) or tracksuits (30.4%, 48) were used for BASE jumping. Eight patients performed regular “classic” BASE jumping without suits. The jumping technique was unknown in 19.6% (31) of the cases.

Information about prior BASE experience was available for 106 (66.7%) patients, of whom 53.8% (57) had made more than 150 and 85.8% (91) more than 50 BASE jumps. For 83 (78.3%) of these, data on previous skydiving experience were also available, showing that 91.7% (76) of the jumpers had made more than 150 skydiving jumps.

Rescue missions were mostly conducted by aerial means (74.1%, 117), with the rest of the missions being terrestrial (21.5%, 34), combined (3.8%, 6), or unknown (1 case). The long-line technique, with a line of over 100 m, was used in 20 (12.7%) jumps, with the longest line being 320 m. The winch technique with helicopter was used in another 19 (12%) jumps, with a maximum length of 75 m.

Between 2007 and 2016, totals of 21 evacuations of uninjured BASE jumpers, 39 fatalities and 98 injuries were recorded.

The number of jumps per year ranged from 5000 to 20,000. The risk of injury was calculated as the number of injuries per number of jumps over the entire period and ranged from 0.05% to 0.2%. The risk of fatality was also based on the range from 5000 to 20,000 jumps per year and ranged from 0.02% to 0.08%.

After exclusion of all patients with NACA 0 or 7, 100 patients with NACA 1–5 were included in the analysis of under- and overtriage; no patient with NACA 6 was found. ISS ranges and confidence intervals (CI) for each NACA category are displayed in Table 1.

Of all patients, 87% (87) had an ISS below 15 (Table 2), while 3.4% (2) of all NACA 1–3 patients had an ISS higher than 15 and 73.2% (30) of all patients with NACA 4–6 had an ISS lower or equal to 15.

All patients with NACA 1 or 2 had an ISS ≤ 15, and were either dispatched to the regional hospital or received out-of-hospital medical assessment, including consultation in Dr. Durrer’s private practice.

Of patients with NACA 3, 95.2% (40) had an ISS ≤ 15. All were initially dispatched and treated in the regional hospital or received out-of-hospital medical treatment. Four patients needed secondary transfer to the level I centre (9.5%), and three needed level I expertise, even though their ISS scores were lower than 15; these patients received a consultation in otolaryngology (ENT), neurosurgery or vascular surgery. One patient had an ISS > 15 associated with multiple trauma and needed specialised pelvic surgery.

Patients with NACA 4 were dispatched to either the regional hospital (54.3%, 19) or the level I centre (45.7%, 16); five patients (14.3%) had a secondary transfer to the level I facility. These patients all had an ISS < 15, with two being transferred due to limited capacity in the operating theatre, and three for specialised orthopaedic care.

Five of six patients with NACA 5 (83.3%) were directly dispatched to the level I centre. One of these had an ISS lower than 15 but suffered from a pelvic ring fracture, one patient died in the ER, and three had an ISS higher than 15 and survived. One patient with NACA 5 was transferred from the regional hospital to the level I facility due to haemodynamic instability and died there. For details, see Table 3.

## 4. Discussion

This retrospective cohort study of all BASE jumping accidents in the valley of Lauterbrunnen between 2007 and 2016 included 158 patients. The design of this study, with access to prehospital (Air Glaciers) and clinical information (general practitioner, two hospitals), and from 50,000 to 200,000 jumps over a period of 10 years, makes it, to our knowledge, the most comprehensive study on BASE jumping.

Our patients were typically male (93%) and young (mean age 34.3). They predominantly used wingsuits (44.9%) or tracksuits (30.4%). Most of them were experienced skydivers: most of the 106 patients with information about their experience had more than 150 skydiving jumps, which, although not a formal rule, is usually considered as the minimum for most first BASE jump courses [21,22]. More than 80% had performed over 50 BASE jumps before the accident. We recorded a total of 21 evacuations, 98 injuries and 39 fatalities in our cohort.

Absolute risks of injury or fatality are very difficult to determine in BASE jumping, as for other extreme sports. Risk is influenced by numerous environmental factors, such as exit difficulty, jumping technique, weather conditions, or peer pressure, which can all influence the risk behaviour.

In this study, we found an injury risk of 0.05–0.20%, which is less than the value reported in previous studies. Soreide’s series from a single jump site in the Kjerag Massif reported a rate of 0.4% [2]. A study by Monasterio and Mei-Dan of 35 experienced BASE jumpers also showed an estimated injury risk of 0.4% [8]. Another more recent study by Mei-Dan covered 68 self-reporting BASE jumpers and showed a rate of 0.2% severe injuries [9]. Soreide et al. in 2012 found an estimated risk in BASE jumping for any injury to be 0.4–0.5%, making BASE jumping five to eight times more dangerous than skydiving [10].

The fatality risk in our study was of 0.02–0.08%. This is similar to the fatality risk of 0.04% observed in Soreide’s study [2]. To our knowledge, only one other study evaluated mortality, but reports this as fatality per BASE jumper in 2002, making comparison difficult [4].

The reduced injury burden could be explained by several factors. The present project is a single-site study with the danger of bias: we might have selected a sub-group of more experienced BASE jumpers who come to the valley of Lauterbrunnen, a region with difficult terrain. Indeed, for patients where information about prior BASE experience was available, 85.8% had more than 50 BASE jumps and 91.7% more than 150 skydiving jumps.

Ongoing technical advancement of the equipment for BASE jumping might be responsible for reduction in the numbers of injuries, and further development of sophisticated rescue techniques might influence survival rates. During the ten years of observation, we saw a reduced injury burden, which may be due in part to technical improvements. Most patients were experienced BASE jumpers, which on the one hand reduces the risk, but on the other hand may increase the willingness to take risks. Meticulous preparation remains one of the keys to preventing accidents and fatalities in BASE jumping.

In our study, undertriage was found in only two cases (3.4%) in the group of patients with NACA 1–3, which is under the threshold considered acceptable by the American College of Surgeons Committee on Trauma (ACS-COT). An undertriage rate of <5% and an overtriage rate of <35% are often considered acceptable, according to the ACS-COT [23]. Both these patients were initially dispatched to the Level II hospital, and one needed transfer to the Level I hospital for specialised surgery. The other patient could be treated conservatively in the regional hospital. The emergency physician and general practitioner of the valley of Lauterbrunnen had many years of clinical experience with injuries specific to BASE jumping [24], and this could have led to this low undertriage rate.

Overtriage was significant, with 73.2% of all NACA 4–6 not qualifying as major trauma. A subgroup analysis showed that overtriage was even higher for patients with NACA 4 (82.9%), but much lower in patients with NACA 5 (16.7%). As BASE jump accidents are usually linked to high morbidity and mortality, this might have influenced emergency physicians to expect the worst, and thus contributed to overtriage. Furthermore, deceleration forces may lead to severe injuries—mainly of the aorta or the hollow viscus organs—even when the trauma has been initially perceived to be blunt and minor [25,26,27]. According to ATLS guidelines, the trauma mechanism must be taken into consideration when assessing a trauma patient, and this might have contributed to defensive triage by the prehospital team.

In general, undertriage is more harmful to the patient. Overtriage, except in the case of a mass event, has little impact on the patient, but rather on the distribution of hospital resources and the cost of care [23].

### Limitations

Despite cross-referencing the unofficial register, the mission reports of the helicopter company and the medical information from the two hospitals, we might have overlooked patients who did not seek medical assistance despite a substantial injury. Data collection might be incomplete due to the retrospective study design. Medical records must be kept only ten years in Switzerland; therefore, clinical records were not available for every patient, especially for patients who were treated in the first years of the study at the private practice of Dr Durrer. This led to a greater number of exclusions, especially for injured patients, introducing a possible selection bias, as the excluded patients with incomplete data sets may have had characteristics differing from the included patients.

After the death of Dr Bruno Durrer in 2016 the unofficial register was not updated. We therefore decided to limit the study to the ten-year period for which the best data were available.

## 5. Conclusions

BASE jumping remains a high-risk sport and is related to significant rates of injuries and fatalities. While a potential reduction of the injury rate was identified in comparison with previous studies, we found no decrease in the fatality rate.

In this well-known BASE jumping environment, prehospital assessment appears to have benefited the patients, as we found a low undertriage rate. On the other hand, the defensive triage associated with high overtriage might, in the face of the increasing numbers of BASE jumpers in the valley of Lauterbrunnen, have had an impact on the future distribution of hospital resources and should be minimised.

The number of jumps per person in the valley of Lauterbrunnen has only recently been recorded, and may in the long run allow a better estimation of mortality and morbidity.

## Figures and Tables

**Table 1:** NACA and ISS.

NACA	Mean ISS	Min. ISS	Max. ISS	CI 95%
1	1.00	1	1	NA
2	3.13	1	14	3.13 ± 3.13
3	5.31	1	19	5.31 ± 1.17
4	10.46	2	25	10.46 ± 1.78
5 ^1^	30.83	10	49	30.83 ± 10.67
Total	8.08	1	49	

^1^ No NACA 6 recorded.

**Table 2:** Under- and overtriage.

	ISS ≤ 15		ISS > 15	
	Number	Percentage	Number	Percentage
NACA 1–3 (n = 59)	57	96.6%	2	3.4%
NACA 4–6 (n = 41)	30	73.2%	1s1	26.8%
Total (n = 100)	87	87%	13	13%

**Table 3:** Numbers of NACA patients according to initial hospital dispatching and to ISS.

NACA	ISS ≤ 15		ISS > 15		
	Number	Percentage	Number	Percentage	
1	9	100.0%	0	0.0%	9
Level II Centre	1	100.0%	0	0.0%	1
No in-hospital assessment	8	100.0%	0	0.0%	8
2	8	100.0%	0	0.0%	8
Level II Centre	1	100.0%	0	0.0%	1
No in-hospital assessment	7	100.0%	0	0.0%	7
3	40	95.2%	2	4.8%	42
Level II Centre	39	95.1%	2	4.9%	41
Stayed in Level II	36	97.3%	1	2.7%	37
Transfer Level II to I	3	75.0%	1	25.0%	4
No in-hospital assessment	1	100.0%	0	0.0%	1
4	29	82.9%	6	17.1%	35
Level I Centre	11	68.8%	5	31.2%	16
Level II Centre	18	94.7%	1	5.3%	19
Stayed in Level II	13	92.9%	1	7.1%	14
Transfer Level II to I	5	100.0%	0	0.0%	5
5	1	16.7%	5	83.3%	6
Level I Centre	1	20.0%	4	80.0%	5
Level II Centre	0	0.0%	1	100.0%	1
Transfer Level II to I	0	0.0%	1	100.0%	1
Total	87	87%	13	13%	100

Level I Centre: University Hospital Bern; Level II Centre: Hospital Interlaken. Transfer from level II to level I Centre is displayed.

## Data Availability

Data supporting reported results are available from the authors.

## List of Abbreviations

ACS-COT	American College of Surgeons Committee on Trauma
ATLS	Advanced Trauma Life Support
AIS	Abbreviated Injury Scale
BFL	BASE Fatality List
BASE	Buildings, Antennas, Spans and Earth
ENT	Ear, Nose & Throat
HEMS	Helicopter Emergency Medical Service
ISS	Injury Severity Score
NACA	National Advisory Committee of Aeronautics
SBA	Swiss Base Association
